# Supplementary figures and images for: Membrane-dependent actin polymerization mediated by the Legionella pneumophila effector protein MavH
Source: PLoS Pathog. 2023 Jul 18;19(7):e1011512. doi: 10.1371/journal.ppat.1011512 (PMC10381072; doi:10.1371/journal.ppat.1011512)

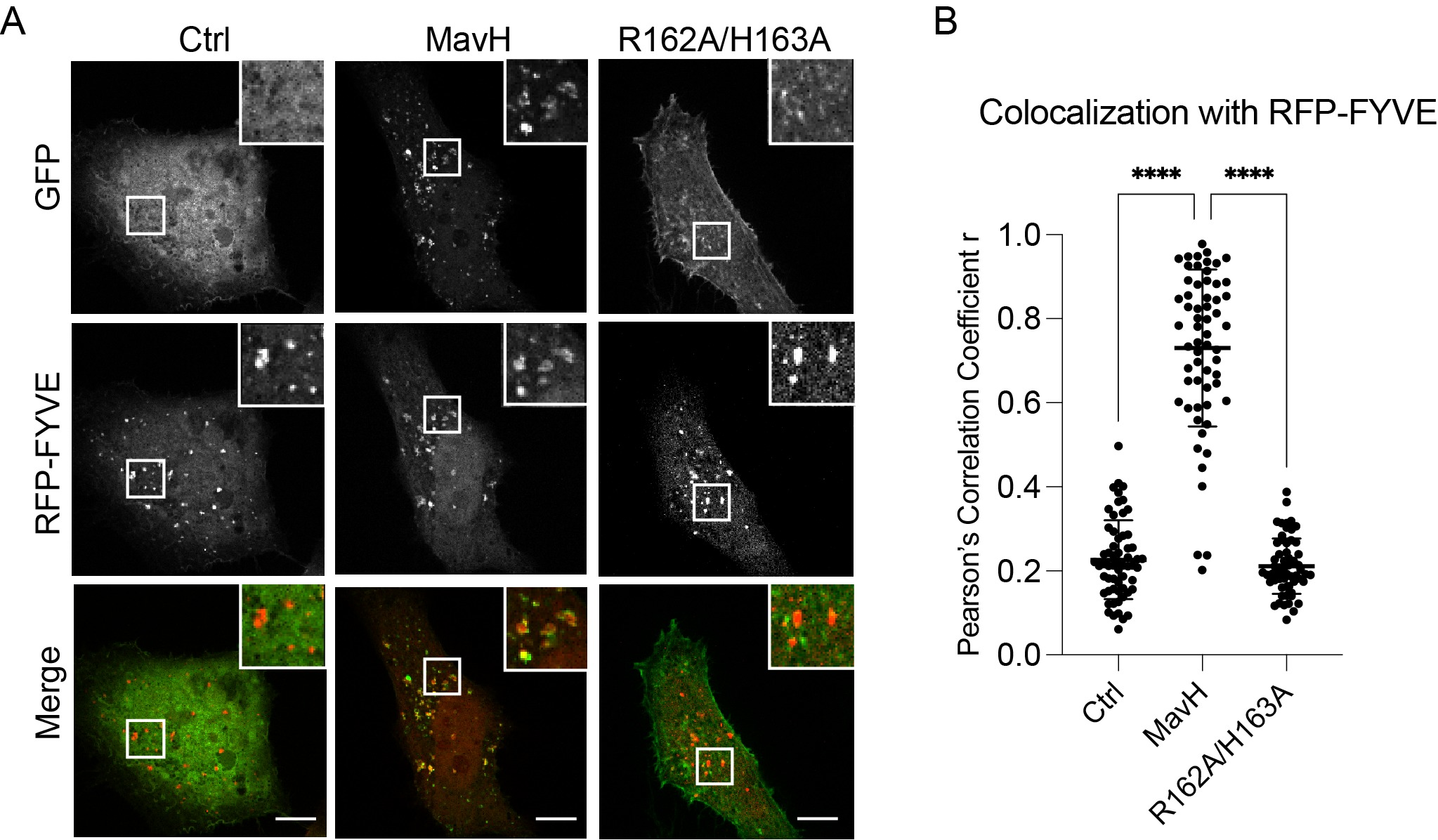

Supplement: S1 Fig — Hela cells were co-transfected with a plasmid expressing RFP-2xFYVE and either with GFP, GFP-MavH, or GFP-MavH R162A/H163A for 20 hours. The colocalization of MavH with the PI(3)P marker RFP-2xFYVE was analyzed by confocal fluorescence microscopy. Wild-type MavH showed a high degree of colocalization with RFP-2xFYVE, while the MavH R162A/H163A mutant defective of PI(3)P-binding exhibited no colocalization with RFP-2xFYVE. Scale bars, 10 μm. (B) Colocalization of GFP fusion proteins with RFP-FYVE. Colocalization was determined by Pearson’s correlation coefficient r. Data are depicted as scatterplots showing mean ±SD from 60 cells in 3 independent experiments. Statistical significance was assessed using one-way ANOVA (****P<0.0001). (TIF) [file ppat.1011512.s001.tif]

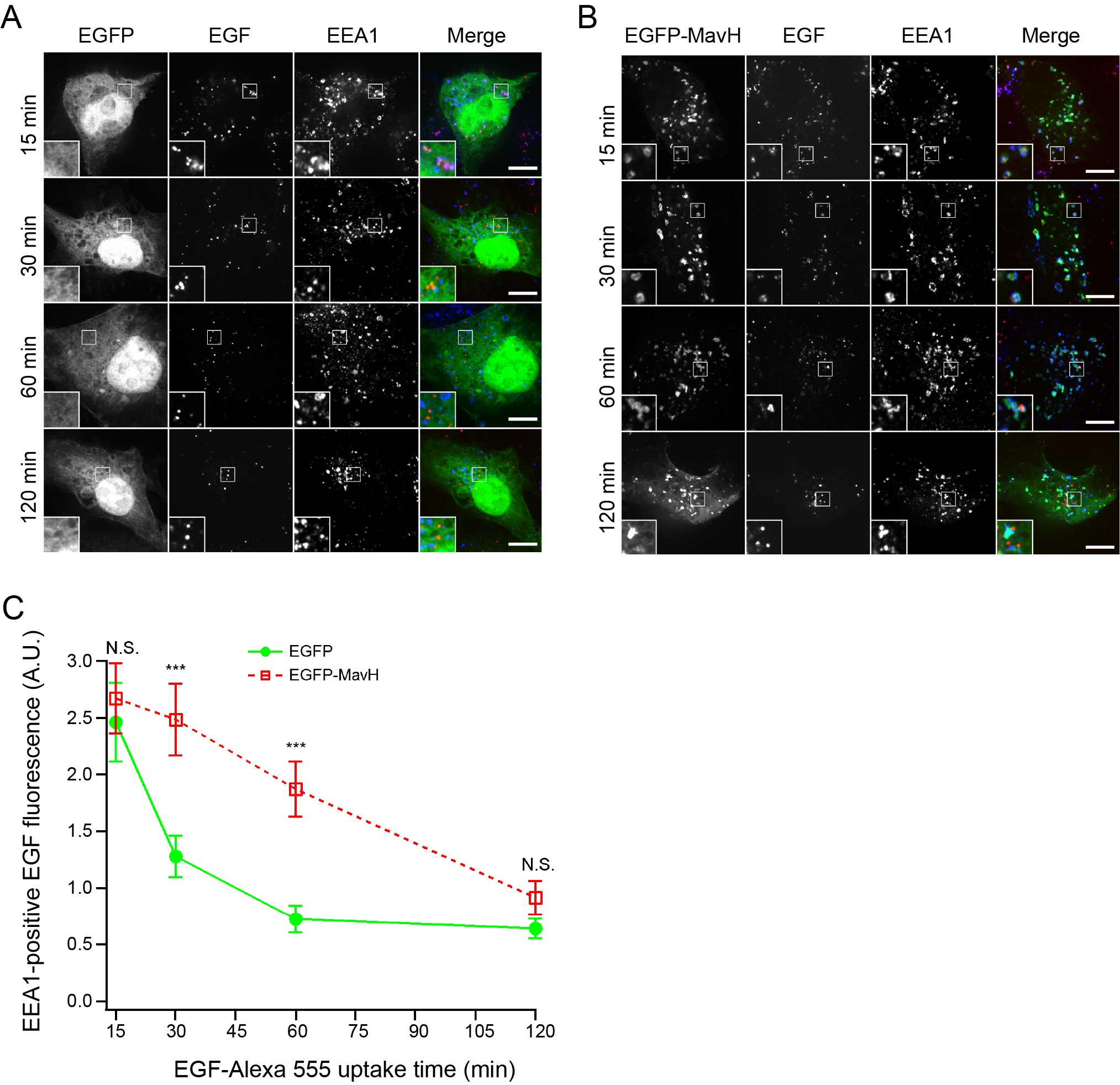

Supplement: S2 Fig — Cos7 cells were first transfected with indicated plasmids for 24 hours. Cells were then incubated with 20 ng/mL Alexa 555-EGF on ice for 20 min, washed, and then incubated at 37°C for the indicated time. Early endosomes were stained with EEA1 antibodies. Representative images are shown for EGFP (A) or EGFP-MavH (B) transfected cells. EGFP-tagged protein is shown in green, Alexa 555-EGF is in red, and EEA1 is in blue. Scale bars are 10 μm. (C) EEA1-positive EGF fluorescence was quantified, shown as Mean ± SEM from three independent experiments. At least 28 cells/conditions were analyzed. ***P<0.001, N.S., not significant. (TIF) [file ppat.1011512.s002.tif]

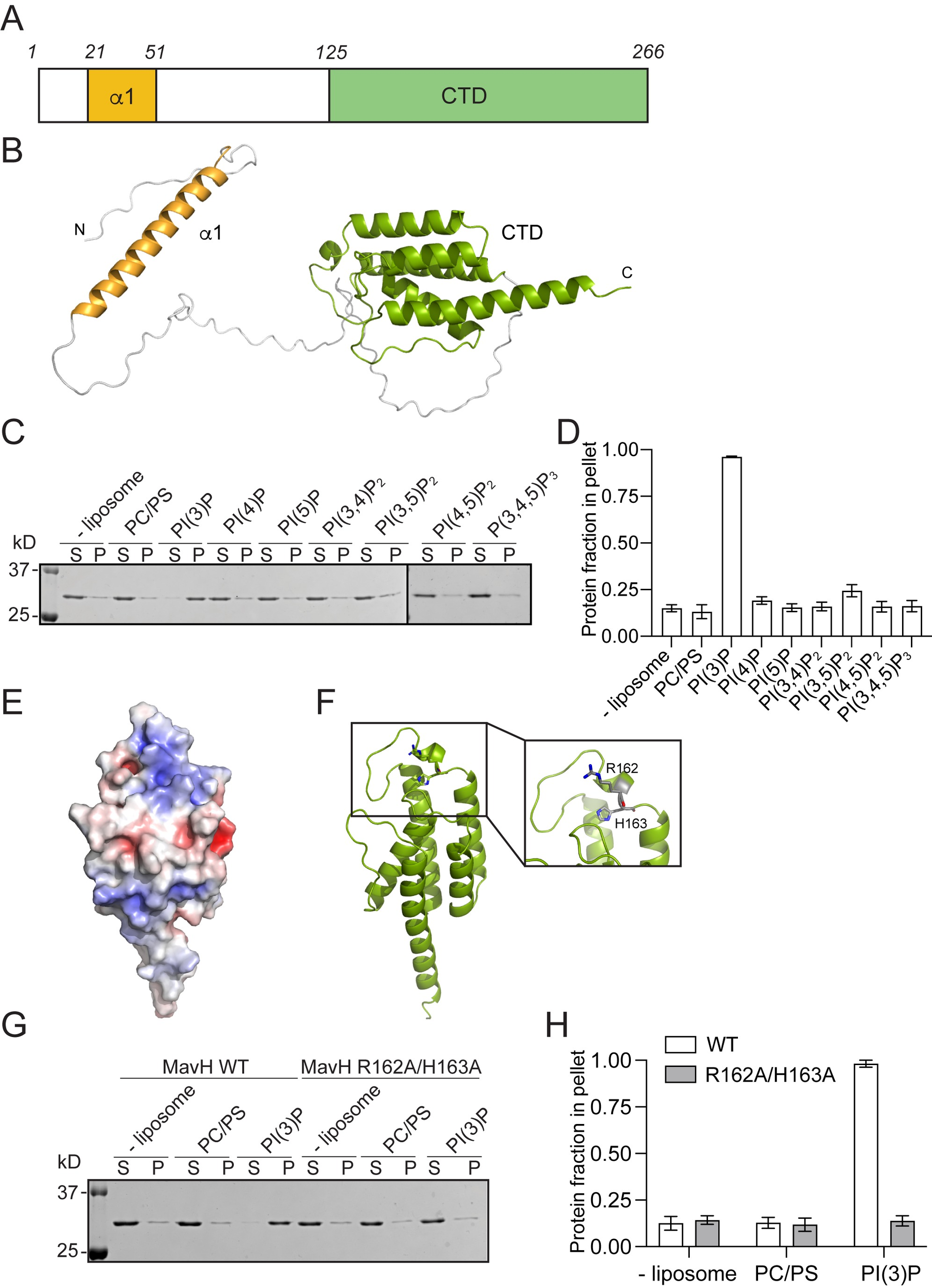

Supplement: S3 Fig — (A) Schematic representation of MavH. N-terminal α-helix (α1) is colored in yellow. The C-terminal domain (CTD) is in green. (B) Ribbon diagram of predicated MavH structure with AlphaFold2. (C) Liposome co-sedimentation assays of MavH. Liposomes were formed with PC, PS, and indicated phosphoinositides. After incubation with MavH, the liposomes were pelleted by ultracentrifugation. P, pellet; S, supernatant. Pellet and supernatant fractions were then analyzed by SDS-PAGE, followed by Coomassie staining. (D) Quantification of the liposome sedimentation assays in (C). The protein factions in the pellet are shown as mean ± SEM from three independent experiments. (E) Molecular surface of CTD of MavH. The surface is colored based on the electrostatic potential with the positively charged region in blue and the negatively charged surface in red. (F) Ribbon representation of CTD of MavH. The conserved positively charged residues, R162 and H163, are shown in sticks. (G) Liposome co-sedimentation assays of MavH wild-type and R162A/H163A mutant. (H) Quantification of liposome sedimentation assays in (G). The protein fractions in the pellet are shown as mean ± SEM from three independent experiments. (TIF) [file ppat.1011512.s003.tif]

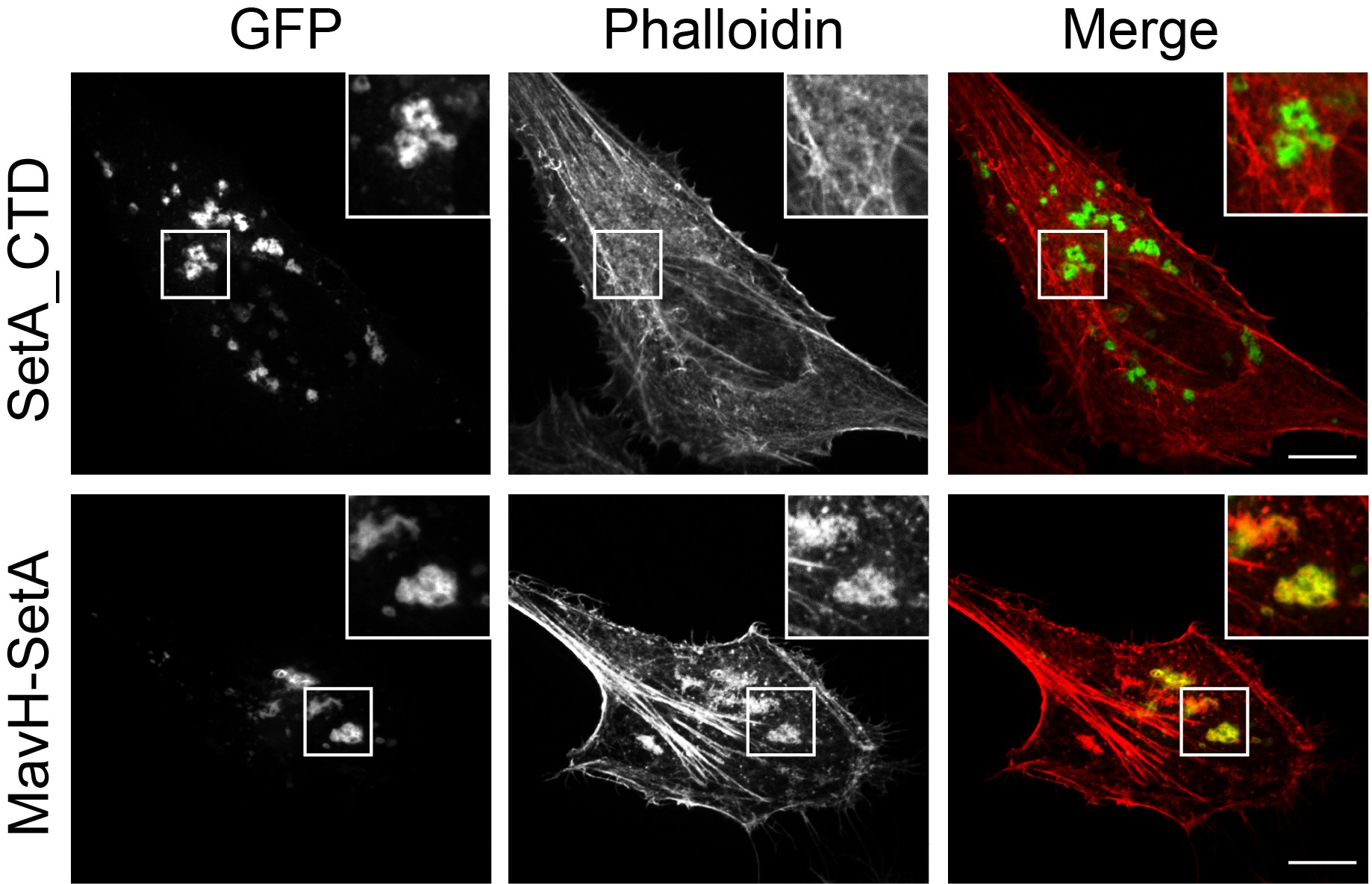

Supplement: S4 Fig — HeLa cells were transfected with GFP-tagged constructs of either SetA PI(3)P-binding domain (SetA_CTD) alone or a chimeric fusion (MavH-SetA) containing the N-terminal portion of MavH in-frame with the SetA_CTD. After transfection with indicated plasmids for 18 h, cells were fixed and stained with Rhodamine conjugated phalloidin and imaged by confocal microscopy. Scale bars are 10 μm. (TIF) [file ppat.1011512.s004.tif]

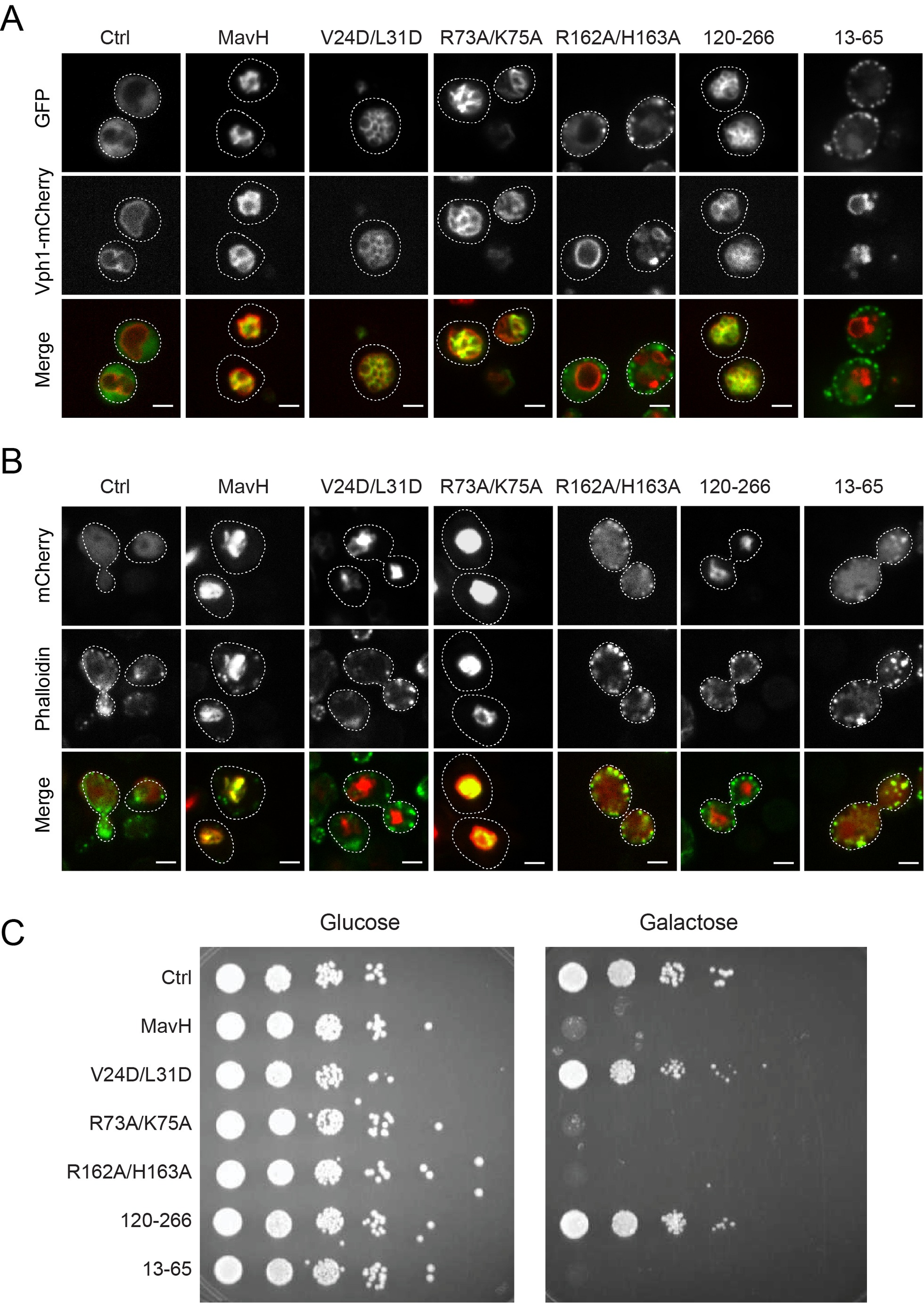

Supplement: S5 Fig — (A) SEY6210.1 yeast strains expressing integrated copies of mCherry-tagged yeast vacuolar marker VPH1 were transformed with either GFP-tagged wild-type MavH, truncations, or mutant constructs under the control of a galactose inducible promoter. Cells were visualized by fluorescence confocal microscopy after induction of protein expression by selective media containing 2% galactose. (B) BY4741 yeast strains were transformed with either mCherry-tagged wild-type or mutant MavH constructs under the control of a galactose inducible promoter. After induction of protein expression by selective media containing 2% galactose, yeast cells were stained with Alexa Fluor 488-phalloidin and visualized by fluorescence confocal microscopy. (C) Yeast cultures from (B) were grown on plates containing glucose or galactose (inducing conditions). 10-fold serial dilutions of each yeast cell culture were spotted on the plate and the lethal effects were compared to the empty vector control. (TIF) [file ppat.1011512.s005.tif]

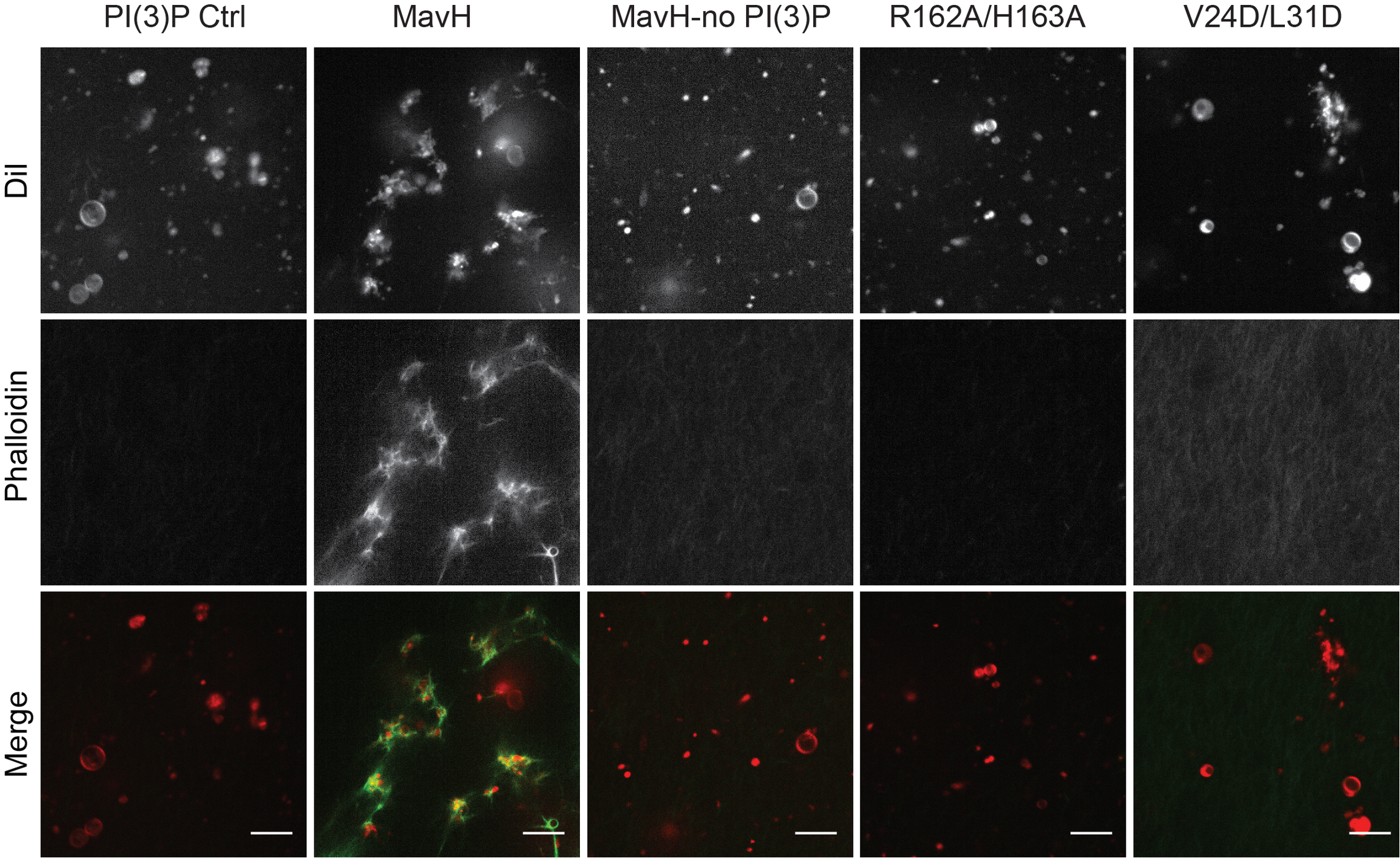

Supplement: S6 Fig — In vitro liposome imaging assay. Reactions were performed using 3 μM actin and 250 nM MavH. Liposomes were used at 500 μM. After induction of actin polymerization, actin was stained with Alexa Fluor 488-phalloidin. Images were taken by confocal microscopy. (TIF) [file ppat.1011512.s006.tif]

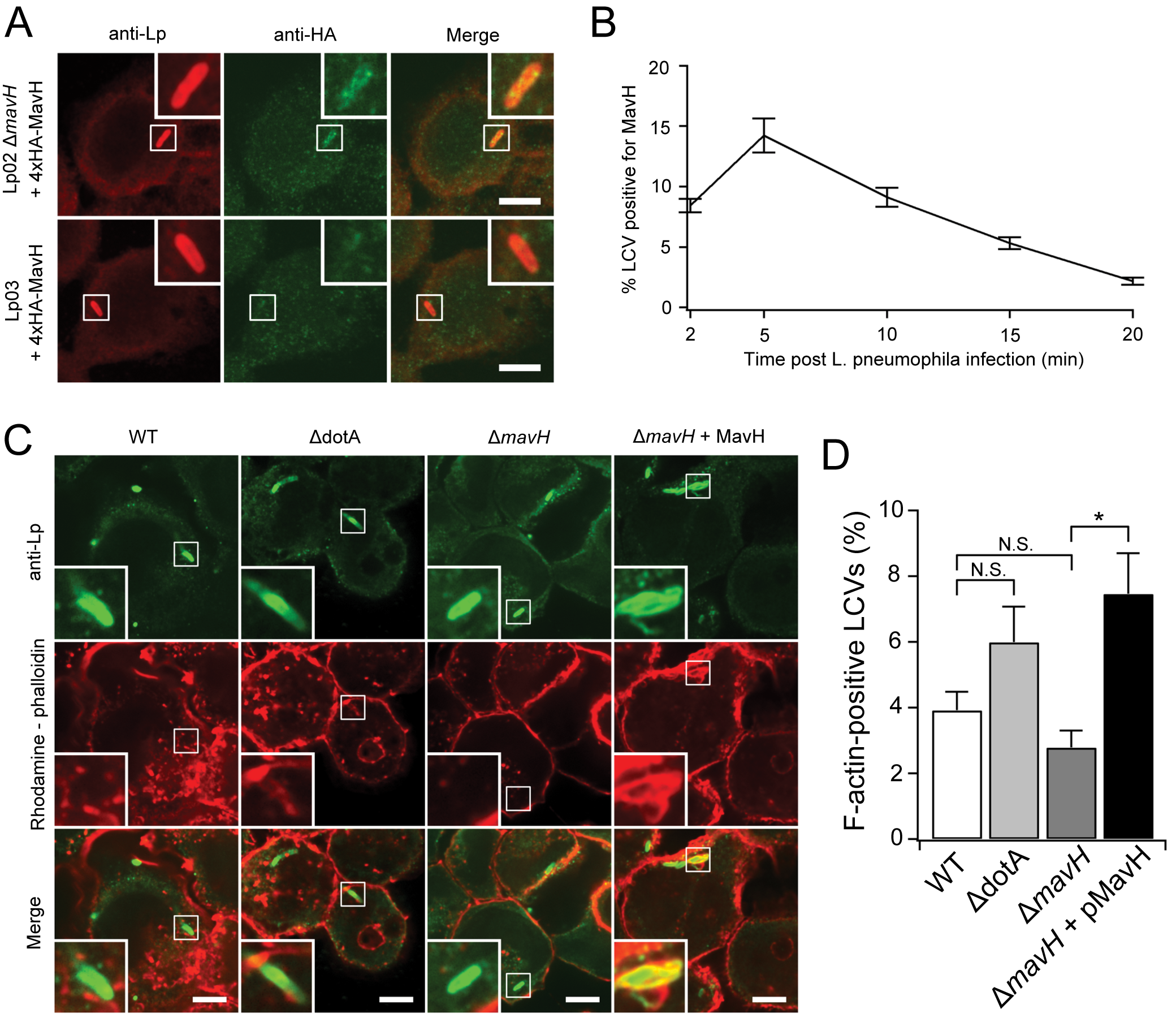

Supplement: S7 Fig — (A) Representative images show localization of 4xHA-MavH at 5 min post-infection. PMA-induced U937 cells were challenged by wild-type ΔmavH or ΔdotA strains supplemented with a plasmid expressing 4xHA-MavH for 5 min. Cells were fixed using 4% PFA for 15 min and then permeabilized using ice-cold methanol for 10 min. 4xHA-MavH was immunostained using mouse-anti-HA primary antibodies and Alexa-488 anti-mouse secondary antibodies. Scale bars are 10 μm. (B) Quantification of LCVs positive for 4xHA-MavH in U937 cells infected by WTΔmavH overexpressing 4xHA-MavH for the indicated time, shown as mean ± SEM from three independent experiments. At least 50 LCVs were analyzed for each time point. (C) Representative images show Legionella bacteria (green) and Rhodanmine-phalloidin-labeled F-actin (red) in PMA-induced U937 cells challenged by the indicated Legionella strains for 10 min. Scale bars are 10 μm. (D) Quantification of LCVs positive for F-actin in U937 cells in (E) shown as mean ± SEM from three independent experiments. At least 50 LCVs were analyzed for each time point. *P < 0.05, N.S. = not significant. (TIF) [file ppat.1011512.s007.tif]
