# Supplementary material for: Membrane-dependent actin polymerization mediated by the Legionella pneumophila effector protein MavH
Source: PLoS Pathog. 2023 Jul 18;19(7):e1011512. doi: 10.1371/journal.ppat.1011512 (PMC10381072; doi:10.1371/journal.ppat.1011512)
Supplement: Attachment — Submitted filename: Rebuttal_Final.docx [file ppat.1011512.s010.docx]

Reviewer's Responses to Questions

**Part I - Summary**
Please use this section to discuss strengths/weaknesses of study, novelty/significance, general execution and scholarship.

Reviewer #1: The study by Zhang et al provides some compelling results to demonstrate that the Legionella pneumophila effector MavH modulates the cytoskeleton of host cells by interacting with capping proteins, promoting actin polymerization in a membrane- (liposome) dependent manner. The study is comprehensive and with a few exceptions, the results are of high quality. The study needs further experiments to address some of the pressing questions in the study of Legionella effectors such as functional redundancy among effectors with similar cell biological impact.

A: We truly appreciate the reviewer’s positive comments. We agree that further experiments are needed to address some of the pressing questions regarding the functional redundancy among *Legionella* effectors. As discussed in the revised text, several *Legionella* effectors have been identified to modulate the actin cytoskeleton through diverse mechanisms. For instance, effectors such as RavK, LegK2, Ceg14, and WipA have been found to inhibit actin polymerization. Conversely, effectors including VipA and MavH (in this study) have been shown to promote actin polymerization. These findings highlight the challenges in deciphering the precise importance of targeting the actin cytoskeleton in *Legionella* infection. We believe each of these actin-hijacking effectors is tightly regulated both temporally and spatially. Future studies are warranted to unravel the holistic actions of these effectors.

Reviewer #2: The study by Zhang et al. investigates the mode of action of the type IV secretion system (T4SS)-translocated effector protein MavH, produced by the causative agent of Legionnaires’ disease, Legionella pneumophila. MavH was identified in a screen for L. pneumophila effectors that perturb the host actin cytoskeleton. Ectopically produced GFP-MavH showed a punctate localization and co-localized with the early endosomal marker EEA1, in agreement with an earlier study documenting the direct binding of MavH to the endosomal phosphoinositide (PI) lipid PI(3)P. The authors then go on to show that (i) MavH recruits host actin capping proteins (CP) and actin to endosomes via its central CP-interacting (CPI) motif and an N-terminal WASP homology (WH2)-like actin binding domain, (ii) MavH inhibits actin polymerization in absence of membranes in vitro, but stimulates actin polymerization in presence of PI(3)P-containing membranes, (iii) MavN recruits CP to negatively regulate actin density at membranes, and (iv) MavN is secreted by the T4SS and localizes to the pathogen vacuole membrane very early during infection.
The manuscript is well-written, and the story unfolds in a straightforward, logical manner. The putative CPI motif and the WH2 domain of MavH were validated by convincing mutagenesis analysis and conclusive cell biological as well as biochemical readouts. Overall, the finding that MavH adopts a novel mechanism to nucleate actin is very intriguing. In contrast to the known actin nucleation mechanisms (Arp2/3 complex, formins, tandem actin-binding domain proteins), a single actin-binding WH2 domain, in concert with PI(3)P-containing membranes, suffices for the effector MavH to nucleate actin. A few rather minor points should be addressed to further strengthen the study.

A. We appreciate the positive comments.

Reviewer #3: The manuscript describes a Legionella effector protein, MavH, that has been characterized in the study by the authors to localize to endosomes via a PI3-binding domain. Furthermore, two N-terminal domains were identified that indicate a potential role of MavH for actin polymerization around endosomes during Legionella infection. These domains are a capping-binding protein binding domain, and also a WH2-like domain for actin binding. In vitro assays were performed that demonstrate actin polymerization dependent on these domains in the presence of PI3P-containing liposomes. Also, pulldowns were performed to characterize the interaction with the proposed partners. These in vitro assays are very convincing. Nevertheless, the cell biological characterization through co-localization is very preliminary, the EM data not interpretable, and the infection assays in figure 6 also rather preliminary. Therefore, I feel that the manuscript has too many “open ends” to allow the conclusions of the authors. Even though well written, a huge amount of additional experiments, analysis and controls would need to be performed to link the “in vitro” data with the proposed role of MavH during infection.

A. Again, we appreciate positive comments about our studies. To address some of the concerns of the reviewer, we have performed a large number of experiments during the revision period and we have obtained a fair amount of new data. Some of the new results include:

1) We performed co-localization assays of MavH/actin/EEA1 (new Figure 1A) and calculated Pearson’s correlation coefficient of the co-localization of MavH with actin, EEA1, or RFP-FYVE (new Figure 1B, C, and S1B).
2) We performed total internal reflection fluorescence microscopy (TIRFM) imaging analysis to show membrane tubulation induced by MavH in real-time (new Figure 4C-F, new Figure 5 and supplemental videos 1 and 2).
3) We also provided EM images of a control condition that lacks PI(3)P to show the membrane tubulation induced by MavH (new Figures 5D and E).
4) We also examined the localization of MavH during *Legionella* infection in differentiated U937 cells, in addition to the HEK293T cells, and verified its association with LCV (new Figure S7A, B).
5) We investigated the impact of MavH on actin assembly during infection and observed a significant increase in F-actin levels on LCV due to MavH (new Figure S7C, D).
6) We also performed phagocytosis assays and demonstrated that MavH enhances the uptake of *Legionella* by macrophages (new Figure 8B).

**Part II – Major Issues: Key Experiments Required for Acceptance**
Please use this section to detail the key new experiments or modifications of existing experiments that should be absolutely required to validate study conclusions.

Generally, there should be no more than 3 such required experiments or major modifications for a "Major Revision" recommendation. If more than 3 experiments are necessary to validate the study conclusions, then you are encouraged to recommend "Reject".

Reviewer #1: 1. The regulation of actin dynamics by MavH appears to be complex in which the CPI motif seems to downregulate actin polymerization by recruiting CP proteins whereas its N-terminal region promotes actin polymerization in the presence of liposome (membrane). The effects on actin polymerization shown in Figure 4 are very modest. Can this be due to the presence of the CPI domain? Although this experiment was done in the absence of CPs, the authors may add experiments using MavH mutants lacking the CPI motif. In the same line of thinking, binding to lipid may expose the structural features in MavH directly involved in promoting actin polymerization. This point should be discussed.

A: Thank you for your comments and suggestions.

1) In the presence of both MavH and PI(3)P-containing liposomes, the absolute value of the fluorescence showed a “modest” change, however, the fluorescence signal exhibited an abnormal fluctuation, which is often observed when the light from a fluorophore is scattered by large particles like filament bundles. The impact of MavH on actin assembly is “huge” as evidenced by our new results from TIRFM microscopy studies, which convincingly demonstrated that MavH promotes actin assembly, inducing membrane tubulation in a PI(3)P and liposome-dependent manner (new Figure 4C, D, E, and F and new Figure 5A, B, and C).

2) Regarding the MavH CPI mutant, we made attempts to express fusion proteins; however, we encountered difficulties as the proteins were unstable and could not be successfully purified for biochemical assays.

3) In the discussion section, we discussed the possibility that binding to lipids may induce structural rearrangement of the protein, which allows the N-terminal WH2 domain to be fully exposed for actin recruitment and assembly.

2. Based on the results, what is the net outcome of the effect of MavH on the accumulation of actin polymers on the LCV? Inhibition or induction? An inhibitory effect would suggest functional redundancy with the activity of RavK, which raised a questions whether a double mutant lacking both mavH and ravK will be detectably defective in intracellular growth. The authors are in a perfect position to examine this hypothesis.

A: Thank you for the insightful discussion. As discussed in the text, several effectors have been shown to interfere with the host actin cytoskeleton, either promoting or inhibiting actin polymerization. In terms of MavH, our in vitro data demonstrated that MavH promotes actin polymerization on PI(3)P-containing membrane and deforms/tabulates the membrane. Our new experiments also revealed the positive correlation of MavH with F-actin levels on Legionella-containing vacuoles (LCVs) in both macrophages and HEK293T cells. Our results demonstrate that MavH promotes actin assembly on LCVs during *Legionella* infection (Figure 7E-F and Figure S7 C-D). Previous study shows RavK directly cleaves actin and hence inhibits actin assembly. We expect that the deletion of RavK may prolong the effects caused by MavH. However, extensive future experiments are needed to dissect the interplay of multiple effectors that interfere with the same host cellular process.

3. Figure 6, the use of the 4xHA tagged MavH to examine the dynamics of LCV association by this protein is problematic due to the potential infection phase-dependent regulation of effector expression and translocation. These results should be done with a strain in which the tagged version is knocked into the chromosome. Nevertheless, the observation that even the protein is expressed by an artificial promoter on a multicopy plasmid, such association still occurred, suggesting the existence of a mechanism that function to remove MavH from the LCV as infection proceeds. This point should be discussed.

A. Thank you for your suggestions. We agree it would be ideal to detect the localization of translocated effectors at their endogenous levels by chromosomal knock-in, it is often barely detectable due to the low abundance of the endogenous protein. Overexpressing the effector protein with a supplemented plasmid has been commonly used as an effective way to track the translocation of the effector. In the revised text, we added the following paragraph to the discussion section: “As PI(3)P is tightly regulated by multiple *Legionella* effectors, it accumulates on LCV within 60 s after uptake and is gradually converted into PI(4)P during LCV maturation. Our results, showing that MavH is observed on LCV during the early stage of *Legionella* infection and gradually tapering as the infection progresses, are in agreement with the role of PI(3)P in the early stages of LCV maturation.”

Reviewer #2: 1) Given that MavH is accumulating on the pathogen vacuole membrane at very early time points post infection (Fig. 6B), and an L. pneumophila mutant lacking MavH does not show an intracellular replication phenotype (Fig. 6E), possible effects of MavH on initial bacterial uptake should be assessed. E.g., determine uptake efficiency with fluorescently labelled wild-type and mavH mutant L. pneumophila by FACS.

A: Thank you for your suggestion. During the revision time, we attempted to use FACS analysis to assess the phagocytosis of *L.pneumophila* by macrophages. However, it has a high and diffused background signal when we tried to immunostain the internalized bacteria. The FACS approach failed to distinguish the background signals from the real bacterial signals. Alternatively, we used the fluorescence microscopy method and directly counted the phagocytosed bacteria (new Figure 8B). Our results showed that the phagocytosis of the WT strain was higher than that of Δ*dotA* strain, indicating the involvement of *Legionella* effectors in phagocytosis. We also found that although the deletion of MavH resulted in a slightly decreased phagocytosis rate by U937 cells compared to the WT strain, the phagocytosis rate was increased significantly upon supplementation with 4xHA-MavH. These data were presented as the new Figure 8B.

Reviewer #3: - None of the data on the fluorescence localization data and the co-localizations has been quantified. Nevertheless, this is crucial for this particular study as there is no data presented that co-localization takes place between MavH, the endosomes AND actin patches (three factor co-localizations). Only two factors are shown to co-localize, and never quantified. It is possible that there is no co-localization between the three factors (endosomes, MavH, and actin patches), for example in some cases MavH could co-localize with the actin patches, and in other cases with endosomes. This would need to be addressed systematically throughout the whole manuscript (especially important for fig 1, 2, 3 and 6, and also the suppl figures).

A. Thank you for the critics. We have reanalyzed the images and revised figures to show the co-localization of MavH, actin, and endosomes (Figure 1A). In addition, we quantified the colocalization of MavH with endosomes, actin, and RFP-FYVE by calculating the Pearson’s correlation coefficient (Figure 1B, 1C, and S1B)

- There is only one figure on Legionella infection (Fig 6), and the chosen host cell type is not the most relevant- it is the HEK293T cell model. All other figures are also in cell types that are not necessarily the best models for Legionella infections. It is possible that there are cell-type specific differences how MavH interacts with different compartments, and this should be addressed in cell models relevant for Legionella infections.

A. To address this concern, we performed parallel infection experiments with differentiated human macrophage U937 cells, which showed similar results as using HEK293T cells. In our infection assays, we examined the localization of MavH during *Legionella* infection using differentiated U937 cells and verified its association with LCV (new Figure S7A, B).

- The fluorescence data in figure 6 does not provide sufficient information on the action of MavH as analyzed in the figures before… are there actin patches somewhere? Where does MavH reprogram host actin? All of this should be analyzed in more detail. Also, a time course would be helpful to understand when MavH host cell reprogramming takes place.

A. It’s a very good point. We have revised it by performing additional experiments to examine F-actin levels on LCV during infection using wild-type, Δ*dotA* mutant, WTΔ*mavH* and WTΔ*mavH* supplemented with 4xHA-MavH strains. We observed a significant increase in F-actin levels on LCV due to MavH during infection of both differentiated human macrophage U937 cells and HEK293T cells (Figure 7 E, F and Figure S7C, D). As MavH is translocated at an early stage, the remodeling of the actin cytoskeleton presumably happens at the same time. A detailed time course of actin remodeling will be hard to examine as phagocytosis is a very fast response.

- The data presented in Fig 4B and C does not fit well together. The effects are gradual in Fig 4B, however they appear “black or white” in Fig 4C. One would respect also gradual differences there.

A: The “apparent discrepancy” between old Figure 4B and 4C is mainly due to the way to detect the signals. In Figure 4B, the absolute value of the fluorescence showed a “modest” change, however, the fluorescence signal exhibited an abnormal fluctuation, which is often observed when the light from a fluorophore is scattered by large particles like filament bundles. The impact of MavH on actin assembly is “huge” as evidenced by our new results from TIRFM microscopy studies, which convincingly demonstrated that MavH promotes actin assembly, inducing membrane tubulation in a PI(3)P and liposome-dependent manner (new Figure 4C, D, E, and F and new Figure 5A, B, and C). In Figure 4C (now Fig. S6), the fluorescence microscopy only detects the signals from clustered actin filaments but not single actin filament. That’s the reason why we see the “black and white” results in Figure 4C.

- The EM data lacks any controls and is not interpretable as such.

A. Very good point. As we responded following the summary of Reviewer #3, we have incorporated the control condition in the revised manuscript. Figure 5D includes the control condition, which consists of liposomes lacking PI(3)P. This allowed us to evaluate the effect of MavH’s association with liposomes on actin assembly (Figure 5D, E). The results indicate that MavH induces actin assembly on PI(3)P containing liposomes, leading to membrane tubulation, as compared to the control condition.

**Part III – Minor Issues: Editorial and Data Presentation Modifications**
Please use this section for editorial suggestions as well as relatively minor modifications of existing data that would enhance clarity.

Reviewer #1: 1. Line 28, from the results in Figure 4, the induction of actin polymerization by MavH even in the presence of liposome is not robust at all.

A: This concern has been addressed above.

2. Lines 47-48, precisely speaking the two references (Hilbi et al., 2001 and Watarai et al., 2001) did NOT show the role of Dot/Icm effectors in bacterial entry nor did the paper of Roy et al., 1998 (the first effector RalF was published in 2004). The authors should cite some more recent and relevant works. There are many to choose from.

A. Thank you for bringing this to our attention. We acknowledge that the two references mentioned highlight the differences in phagocytosis rates between the WT(Lp02) and ∆dotA (Lp03) strains, suggesting the potential involvement of *Legionella* effectors in the bacterial entry. However, they do not specifically identify the effectors responsible for modulating phagocytosis. To address this gap, we have revised the manuscript by incorporating additional citations:

Franco, I.S., Shohdy, N., and Shuman, H.A. (2012). The *Legionella* pneumophila effector VipA is an actin nucleator that alters host cell organelle trafficking. PLoS Pathog *8*, e1002546. 10.1371/journal.ppat.1002546.

Prashar, A., Ortiz, M.E., Lucarelli, S., Barker, E., Tabatabeiyazdi, Z., Shamoun, F., Raju, D., Antonescu, C., Guyard, C., and Terebiznik, M.R. (2018). Small Rho GTPases and the Effector VipA Mediate the Invasion of Epithelial Cells by Filamentous *Legionella* pneumophila. Front Cell Infect Microbiol *8*, 133. 10.3389/fcimb.2018.00133.

3. Line 166 will MavHΔN93 be better?

A: Thank you for your suggestion. We have revised the naming of the construct in both the text and the figures accordingly.

4. Line 178, please specify which mutants.

A: Thank you for your suggestion. We revised that part as: “We expressed GFP-tagged wild-type MavH, the PI(3)P-binding domain mutant (MavH R162A/H163A), and the tentative WH2 domain mutant (MavH 24D/L31D) in HEK293T cells and assessed the interaction between MavH and actin by co-immunoprecipitation.”

Reviewer #2: 1) Fig. S1: The co-localization of MavH and RFP-FYVE should be quantified. E.g., calculate Pearson correlation coefficients.

A. Thank you for pointing it out. We have now included the quantification (Pearson's correlation coefficient) of the colocalization between MavH and RFP-FYVE (Figure S1B).

2) Abstract: the statement “In this study, we report that the Legionella MavH effector harbors a lipid binding domain …” should be phrased more precisely, as in fact, a previous study already showed that MavH binds to PI(3)P (Nachmias et al., 2019), and in the current study, no biochemical PI lipid binding assays were performed.

A. Thank you for the comment. We have rephrased it in the manuscript. In the previous study, MavH's binding to PI(3)P was demonstrated through protein-lipid assays. In our current study, we further investigated this interaction by performing a liposome co-sedimentation assay, which confirmed MavH's preference for PI(3)P. Moreover, based on structural analysis, we identified key residues involved in the binding interaction and verified their significance through mutagenesis experiments.

3) The concept of “capping activity” is introduced without further explanation (l. 260), and Fig. 5C shows an in vitro actin polymerization assay, rather than a “capping assay”. This should be rephrased and/or explained for the non-specialist reader.

A. Thank you for your suggestion. We have rephrased it in the manuscript (Figure 6C). Pyrene actin polymerization assay is one of the methods to show inhibitory effect on actin assembly by capping proteins.

4) Fig. 6B: The kinetics of MavH recruitment to the pathogen vacuole membrane formed in HEK293T cells is very fast (minutes). Were the bacteria spun onto the cells, or just left to infect the cells without centrifugation? Were other, more natural host cells tested (amoeba, macrophages)?

A. It’s a very good point. In this manuscript, we spun the bacteria onto the cells to synchronize infection. Reviewer#3 also suggested to use macrophages for infection. In the revised manuscript, we have examined both HEK293T cells and differentiated human macrophage U937 cells, and we observed similar results that MavH is translocated at early stage during infection and associates with LCV.

5) Typos:
- l. 64: … Spire (Kerkhoff, 2006) and Cobl (Ahuja et al., 2007) …
- l. 97: … MavH, which localizes …
- l. 195: … domain from the Legionella effector SetA, …
- l. 777: … dependent on the CPI motif.
- Some references are incomplete/incorrect (e.g., l. 701, 706, 711, 714, 742) and not uniformly formatted.
- Fig. 6B (X-axis: L. pneumophila), Fig. 6E (X-axis: Time (hr)).

A. We are grateful for the comments, and we have corrected all the above grammar/typo mistakes in the revised manuscript.

Reviewer #3: Minor points:
- In figure 2C, the band at around 40kDa should be explained.

A. Thank you for pointing it out. MavH shows several degradation bands when expressed in mammalian cells. We have explained it in the figure legends.

- The IPs lack a control for the overall protein input (could be a house keeping protein).

A. We have added β tubulin as the control in Figure 2C and 3C.

- Fig 4B: why is the MavH signal so jumpy?

A. This comment has been addressed (see above).
